# Acute Toxic Impact of Cyclophosphamide on the Metabolic Organs of Siamese Fighting Fish (*Betta splendens*)

**DOI:** 10.3390/toxics14080700

**Published:** 2026-08-07

**Authors:** Somkiat Sreebun, Sukumal Prukudom, Kannika Siripattarapravat, Supreeya Srisampan, Aksorn Saengtienchai, Piyaporn Eiamcharoen, Santi Poungcharean, Chonphoom Phanpoe, Onanong Suksao, Usuma Jermnak

**Affiliations:** 1 Graduate Program in Animal Health and Biomedical Sciences, Faculty of Veterinary Medicine, Kasetsart University, Bangkok 10900, Thailand; sreebun.s@gmail.com; 2 Department of Anatomy, Faculty of Veterinary Medicine, Kasetsart University, Bangkok 10900, Thailand; sukumal.pru@ku.th; 3 Department of Pathology, Faculty of Veterinary Medicine, Kasetsart University, Bangkok 10900, Thailand; fvetkks@ku.ac.th (K.S.); piyaporn.e@ku.th (P.E.); 4 Center for Veterinary Diagnostic Laboratory, Bangkhen, Faculty of Veterinary Medicine, Kasetsart University, Bangkok 10900, Thailand; yyungvet@gmail.com (S.S.); chonophoom12@gmail.com (C.P.); 5 Department of Pharmacology, Faculty of Veterinary Medicine, Kasetsart University, Bangkok 10900, Thailand; aksorn.c@ku.th; 6 Department of Fishery Biology, Faculty of Fisheries, Kasetsart University, Bangkok 10900, Thailand; ffisstpr@ku.ac.th; 7 Nakhon Pathom Provincial Public Health Office, Nakhon Pathom 73000, Thailand; nuinuinuiyaya@gmail.com

**Keywords:** acute toxicity, cyclophosphamide, Siamese fighting fish, *Betta splendens*, liver, kidney, oxidative stress, emerging pharmaceutical contaminants

## Abstract

Currently, emerging pharmaceutical contaminants (EPCs) pose a significant risk to aquatic biodiversity on both a global and regional scale. Their accumulation in the environment presents several challenges to both human health and ecological integrity. Among EPCs, cyclophosphamide (COP), a widely used alkylating cytotoxic and immunosuppressive drug in human and veterinary oncology, has become one of the most frequently detected environmental contaminants. It has been reported to impair the immune system, induce oxidative stress, and exhibit genotoxic and cytotoxic effects in various fish species. However, limited research has evaluated its toxicity in the Siamese fighting fish (*Betta splendens*), an endemic significant aquatic species in Thailand. This study evaluated the acute toxicity of COP in *Betta splendens* by assessing systemic oxidative stress responses, alterations in gene expression, and localized histopathological changes within both hepatic and renal tissues. The 96 h of median lethal concentration (LC_50_) value for COP in *Betta splendens* was determined to be 793.4 mg/L. High-concentration exposure (800 mg/L) induced severe systemic oxidative stress, evidenced by a significant reduction in superoxide dismutase (SOD) activity and a marked elevation in malondialdehyde (MDA) levels across both liver and kidney. At the transcriptomic level, acute COP exposure significantly upregulated pro-inflammatory (interleukin-1β, *IL*-1*β* and tumor necrosis factor, *TNF-α*), cellular stress (heat shock protein 70, *HSP*70), and apoptotic (caspase-3, *Casp*3) genes. Semi-quantitative histopathological evaluation revealed severe concentration-dependent structural damage. Hepatic lesions peaked at 800 mg/L characterized by diffuse vacuolation, vascular congestion, and extensive necrosis. Similarly, severe renal damage occurred at 800 mg/L, featuring marked vascular congestion, melanomacrophage aggregation, and widespread tubular necrosis. Overall, these findings demonstrate that acute waterborne COP exposure induces severe hepatotoxicity and nephrotoxicity in *Betta splendens* driven by oxidative stress, pro-inflammatory signaling, and apoptotic pathways. This study provides essential baseline toxicity thresholds and highlights the physiological risks COP spills pose to tropical freshwater labyrinth fish.

## 1. Introduction

Pharmaceutical contaminants in the environment represent an emerging and critical threat to biodiversity within both global and local aquatic ecosystems. In fact, this growing contamination has been named one of the top ten emerging issues defining a new direction for global health security [1]. The escalation in global drug usage significantly increases the risk of pharmaceutical residues entering natural aquatic systems. Many of these compounds, particularly anticancer agents, are not completely removed by standard wastewater treatment plants. Consequently, they pose a serious threat to aquatic ecosystems and organisms. Ultimately, human consumption of contaminated water and aquatic species could serve as an exposure route to these pollutants, potentially raising public health concerns. Among these contaminants, cyclophosphamide (COP), a widely used cytotoxic and immunosuppressive drug in human and veterinary oncology [2,3,4] has become one of the most detected environmental contaminants. It is frequently detected globally in various aquatic environments, including hospital effluents, municipal wastewater, and river waters [5,6]. For instance, COP concentrations of 687, 22 and 5.73 μg/L have been reported in the hospital wastewaters in France, Slovenia, and Spain, respectively [7,8,9]. In Thailand, COP has been detected in surface water from the Chao Phraya River at 2 µg/L [10] and in municipal wastewater across Bangkok at concentrations up to 2.06 µg/L [11]. Therefore, the potential ecotoxicological risks to non-target aquatic organisms cannot be overlooked.

Several reports demonstrate that COP impairs the immune system, induces oxidative stress, and exhibits genotoxic and cytotoxic effects in various fish species. For instance, COP has a severe cytotoxic effect on peripheral blood leukocytes of blunt snout bream (*Megalobrama amblycephala*) at a concentration of 0.32–3.2 mg/mL [12] and an immunosuppressed model in Nile tilapia (*Oreochromis niloticus*) can be induced via intraperitoneal COP injection at 200 mg/kg [13]. Mechanistically, COP exposure triggers an acute oxidative burst, leading to an overproduction of reactive oxygen species (ROS) that can overwhelm the host’s natural enzymatic antioxidant defense network [14]. Under normal physiological conditions, superoxide dismutase (SOD) serves as the primary line of defense by converting toxic superoxide anions into hydrogen peroxide. Subsequently, hydrogen peroxide is neutralized into water and oxygen by downstream antioxidant enzymes, including catalase, glutathione peroxidase, and peroxiredoxins. However, excessive COP accumulation disrupts this coordinated enzymatic clearance system, leading to unmitigated hydroxyl radical formation and systemic lipid peroxidation that physically degrades cell membranes across critical metabolic organs such as the liver and kidney. Beyond direct membrane damage, oxidative stress in aquatic species engages broader regulatory signaling networks that coordinate immune responses and metabolic stability. Redox homeostasis is primarily governed by the Nrf2-Keap1 signaling switch, which regulates the transcription of key antioxidant and cytoprotective genes, such as *SOD* and heat shock protein 70 (*HSP*70). Concurrently, ROS accumulation stimulates the MAPK/JNK–NF-κB cascade and activates nuclear factor kappa B subunit 1 (*NF-κB*1) to drive the production of pro-inflammatory cytokines including interleukin-1 beta (*IL*-1*β*) and tumor necrosis factor alpha (*TNF-α*). In parallel, this redox imbalance activates programmed cell death via downstream caspase-3 (*Casp*3)-mediated apoptosis [15,16]. In larval zebrafish, developmental studies reveal that COP exposure significantly reduces survival rates at 5 µM and induces morphological deformities above 1 µM [17,18]. Furthermore, chronic exposure to environmentally relevant COP concentrations causes persistent oxidative stress, metabolic disruption, and ion-regulatory imbalances in zebrafish gills and liver, ultimately leading to dose- and time-dependent tissue damage that parallels mammalian toxicological pathways [19].

The Siamese fighting fish (*Betta splendens*) is representative as a national aquatic animal of Thailand, a globally domesticated ornamental fish species of immense cultural and economic export value. In addition to its commercial significance, the genus Betta serves as an excellent model for studying domestication, social behaviors, and population genetics [20]. This potential is further supported by available of annotated reference genomes for wild and domesticated strains, which enable powerful genomic analyses [21,22]. Because of this long history of breeding, there is now a huge variety of Betta fish available, ranging from original wild strains to colorful, selectively bred domestic varieties [23]. Interestingly, all of these freshwater Betta species share a unique physiological capacity to survive harsh environmental extremes, such as low oxygen and high ammonia [24]. However, human activities such as urbanization, deforestation, and agricultural runoffs are increasingly damaging these native tropical habitats and populations [25]. In particular, growing urban areas frequently release pharmaceutical contaminants into tropical waterways, causing major changes in aquatic ecosystems and a loss of biodiversity [26,27].

Although the toxicity of COP has been investigated in various fish species, the specific toxicological effects of COP in the metabolic organs of the *Betta splendens* remain poorly understood. As a globally popular ornamental species native to Thailand, *Betta splendens* holds both cultural and ecological significance. Consequently, it serves as a highly relevant model for assessing the impacts of emerging chemical stressors in freshwater ecosystems. To better understand these risks, this study evaluates the acute toxicity of COP in *Betta splendens*, demonstrating that it targets key metabolic organs to cause severe cellular damage. Specifically, we evaluated systemic oxidative stress by measuring SOD activity and MDA levels as key indicators of enzymatic antioxidant response and lipid peroxidation damage. To elucidate the underlying molecular mechanisms, we analyzed the gene expression of key targets involved in inflammation (*IL*-1*β, TNF-α*, and *NF-κB*1), cellular stress and antioxidant defense (*HSP*70 and *SOD*), and apoptosis (*Casp*3). These molecular responses, combined with localized histopathological evaluations in the liver and kidney, provide a comprehensive assessment of COP-mediated toxicological impacts in *Betta splendens*.

## 2. Materials and Methods

### 2.1. Ethical Approval

All experimental procedures were approved by the Institutional Animal Care and Use Committee (IACUC), Kasetsart University (Approval No. ACKU68-VET-024), and by the Ethical Review Board of the office of the National Research Council of Thailand (NRCT license No. U1-00477-2558). The acute toxicity test for adult fish was conducted in accordance with OECD Test Guidelines [28]. Fish euthanasia for sample collection was done according to the most recent guidelines of AVMA [29].

### 2.2. Reagents and Chemicals

COP was purchased from Baxter Oncology GmbH (Baxter Oncology GmbH, Halle, Germany). Deionized distilled water was prepared in the laboratory using the Milli-Q purification system (Millipore, Bedford, MA, USA). All solvents and other materials of high-performance liquid chromatography (HPLC) grade were purchased from Tokyo Chemical Industry Co., Ltd. (TCI, Tokyo, Japan).

### 2.3. Experimental Fish

A total of 120 healthy male *Betta splendens* (average 1.83 ± 0.27 g in weight and 5.46 ± 0.15 cm in length) were sourced from an ornamental fish market in Bangkok, Thailand. The fish were acclimated for 21 days in individual glass tanks (7.62 × 10.16 × 17.78 cm) holding roughly 0.8 L of dechlorinated water. No aeration was used during the acclimation and experiment since *Betta splendens* are air-breathing fish. Individual containers were isolated using white paper dividers to eliminate visual contact. During the acclimatization period, the fish were fed artemia twice daily at 5% of their body weight and maintained under a 12:12 h light/dark photoperiod. Daily monitoring confirmed stable water parameters: temperature (26–28 °C) and pH (7.0–7.4) measured via a Pro Smart Sensor 818 pH meter (Smart Sensor, Dongguan, China), alongside dissolved oxygen (8–11 mg/L) and total ammonia (0–0.25 mg/L) assessed with colorimetric test kits (Tetra GmbH, Melle, Germany). Visual health checks confirmed all specimens were free of clinical illness and injuries prior to experimental setup.

### 2.4. Experimental Design

One gram of a stock solution of COP was prepared and diluted immediately prior to each exposure. The test concentrations (0–800 mg/L) for the acute toxicity test were determined based on a review of existing median lethal concentration (LC_50_) data for embryo and larva zebrafish (*Danio rerio*) [18,30] and our preliminary range-finding experiments, a geometric progression series with a spacing factor of 2.0, were established. Five treatments (0, 100, 200, 400, and 800 mg/L) were established in individual 1 L glass tanks containing 0.8 L of dechlorinated water. The design utilized a triplicate setup for each treatment, with 8 fish per replicate. The 96 h experiment was conducted as a static test and the fish were not fed. Fish mortality was monitored twice daily. Concurrently, 20 mL water samples were collected from each glass tank at 0 and 96 h to confirm the actual COP concentrations during the exposure.

### 2.5. Assessment of Total Protein, Antioxidant Enzyme and Lipid Peroxidation Activities in Liver and Kidney

To evaluate the balance between cellular antioxidant defense and oxidative organ damage following COP exposure, superoxide dismutase (SOD) activity and malondialdehyde (MDA) levels were measured in liver and kidney tissues. SOD was selected as a key biomarker for primary enzymatic antioxidant defense, reflecting the tissue’s functional capacity to scavenge toxic superoxide radicals. MDA was chosen as a sensitive marker for lipid peroxidation to directly quantify reactive oxygen species (ROS)-mediated structural degradation of cellular membranes. Tissue samples from liver and kidney (30 mg) were homogenized in 500 µL of chilled sucrose buffer (0.25 M sucrose, 10 mM Tris, 1 mM EDTA, pH 7.4) using a Teflon homogenizer at 4 °C. The homogenates were then centrifuged at 10,000× *g* for 60 min at 4 °C. After centrifugation, the supernatant was harvested for total protein determination via a Pierce BCA protein assay kit (Thermo Fisher Scientific, Grand Island, NY, USA). Total SOD activity was quantified using a WST-based SOD Assay Kit (Dojindo Laboratories, Kumamoto, Japan) by recording absorbance at 450 nm on a BioTek Synergy H1 microplate reader (Agilent Technologies, Winooski, VT, USA). One unit of SOD activity was defined as the amount of enzyme causing a 50% inhibition of WST-1 photochemical reduction. Final SOD activity was normalized to total protein content and expressed as U/mg protein. For lipid peroxidation assessment, MDA levels were evaluated with an MDA Colorimetric Assay Kit (Elabscience Biotechnology Inc., Wuhan, China). Briefly, weighed 30 mg portions of liver and kidney tissue were processed in 300 µL of ice-cold PBS using a Teflon homogenizer at 4 °C, then clarified by centrifugation at 10,000× *g* for 5 min at 4 °C. Supernatants were collected, and MDA content was determined based on the kit’s standard curve according to the manufacturer’s directions.

### 2.6. Expression of Genes Related to Metabolic Stress Response in Targeted Organs

Tissue samples (liver and kidney) of approximately 10 mg were flash-frozen in liquid nitrogen and stored at −80 °C prior to RNA isolation. Total RNA was isolated using the GeneJet RNA Purification Kit (Thermo Fisher Scientific, Waltham, MA, USA) according to manufacturer directions. Genomic DNA contamination was removed by incubating RNA extracts with 1 μL of RQ1 RNase-Free DNase (Promega, Madison, WI, USA) for 20 min. Reverse transcription of 1 µg total RNA into cDNA was performed with the SuperScript^®^ III First-Strand Synthesis Kit (Invitrogen, Carlsbad, CA, USA; Cat# 18080051). Briefly, reactions (20 µL final volume) contained oligo (dT) primers (50 µM), 10 mM dNTP mix, and RNase-free water. Reverse transcription was run on a G-Storm GS482 thermal cycler (Gene Technologies, Somerset, UK) under the thermal regime: 65 °C (5 min), 50 °C (50 min), 85 °C (5 min), and 37 °C for (5 min). To evaluate the toxic impact of COP at the transcriptomic levels, a targeted panel of gene markers representing major pathways of metabolic organ toxicity in liver and kidney was selected. *IL*-1*β, TNFα*, and *NF-κB*1 were selected as primary mediators of inflammatory response and immune dysregulation. *HSP*70 was chosen as a sensitive marker for cellular stress response. *SOD* was evaluated as a first-line enzymatic defense against ROS. *Casp*3 was quantified as a key executioner biomarker for programmed cell death. Transcript levels were quantified using quantitative real-time PCR (qRT-PCR) and normalized to the internal control *β-actin*. *β-actin* was selected as the reference gene due to its demonstrated transcriptional stability in metabolic tissues across toxicological treatments. Preliminary evaluations confirmed that *β-actin* expression remained stable (Cq variation < 0.5 cycles) across control and COP-exposure liver and kidney samples. The qRT-PCR reactions contained 2 μL of cDNA, 10 μL of iTaq Universal SYBR Green Supermix (Bio-Rad, Hercules, CA, USA), and 2 μL of forward and reverse primers (Table 1). Amplifications were performed in five replicates on a CFX96 Touch real-time PCR detection system (Bio-Rad Laboratories, Hercules, CA, USA) using the following profile: initial denaturation at 94 °C (15 min), followed by 40 cycles of 94 °C (15 s), 60 °C (30 s), and 72 °C (1 min). Specificity was verified using melt-curve analysis (65–95 °C). Relative gene expression was computed using the 2^–∆∆ Cq^ method.

### 2.7. Histopathological Examination

Major metabolic organs including liver, and kidney, were immediately collected and fixed in Dietrich’s fixative for 24 h. Post-fixation, tissues were thoroughly washed with distilled water to remove excess fixative and stored in 70% ethanol before being processed for histology. Tissues were dehydrated through a graded ethanol series followed by clearing in xylene. They were then infiltrated and embedded in paraffin wax at 60 °C. The paraffin was changed every 30 min to ensure complete infiltration. The resulting paraffin blocks were sectioned at 5 µm thickness using a rotary microtome. Sections were subsequently stained with Hematoxylin and Eosin and examined at 40× magnification (Nikon Eclipse Ci-L equipped with a DS-Fi2 camera, Tokyo, Japan). The histopathological alterations including vacuolar degeneration, melanomacrophage (MMC) aggregation, blood congestion, and necrosis were semi-quantitatively evaluated across ten randomly selected sections per fish (*n* = 5 fish per group). Tissue damage was evaluated semi-quantitatively using a standardized lesion score (0 = no lesion, 1 = mild, 2 = moderate, and 3 = severe tissue involvement).

### 2.8. Determination of COP Concentrations in Experimental Water

The concentration of COP in experimental water was determined using high-performance liquid chromatography–tandem mass spectrometry (LC-MS/MS). The procedures for sample extraction, LC-MS/MS condition, and method validation were previously described by Saengtienchai et al. [11,33].

### 2.9. Statistical Analysis

All quantitative data were evaluated for normality using the Shapiro–Wilk test. Parametric data (gene expression levels) were presented as means ± standard deviation (SD) (*n* = 5 per group) and analyzed using one-way Analysis of Variance (ANOVA) followed by Dunnett’s multiple comparisons test against the control. Non-parametric data (oxidative stress biomarkers and histopathological lesion score) were analyzed using the non-parametric Kruskal–Wallis test followed by Dunn’s post hoc test. To evaluate dose-dependent trends across groups, a post hoc test for linear trend (*p_trend_*) was performed. For the acute toxicity assay, 24 fish per treatment group (*n* = 24) across 5 exposure concentrations (0, 100, 200, 400, and 800 mg/L) were evaluated. The 96 h LC_50_ value and its 95% confidence interval were calculated using non-linear regression analysis. Mortality data were fitted to a non-linear four-parameter logistic dose–response model (dose vs. response with variable slope). Parameter constraints were fixed at bottom = 0% and top = 100% to reflect full baseline to maximum mortality bounds. All statistical analyses were conducted using GraphPad Prism 8.0 (GraphPad Software Inc., San Diego, CA, USA). Differences were considered statistically significant at * *p* < 0.05, ** *p* < 0.01, and *** *p* < 0.001.

## 3. Results

### 3.1. Acute Toxicity Test

**Figure 1:**
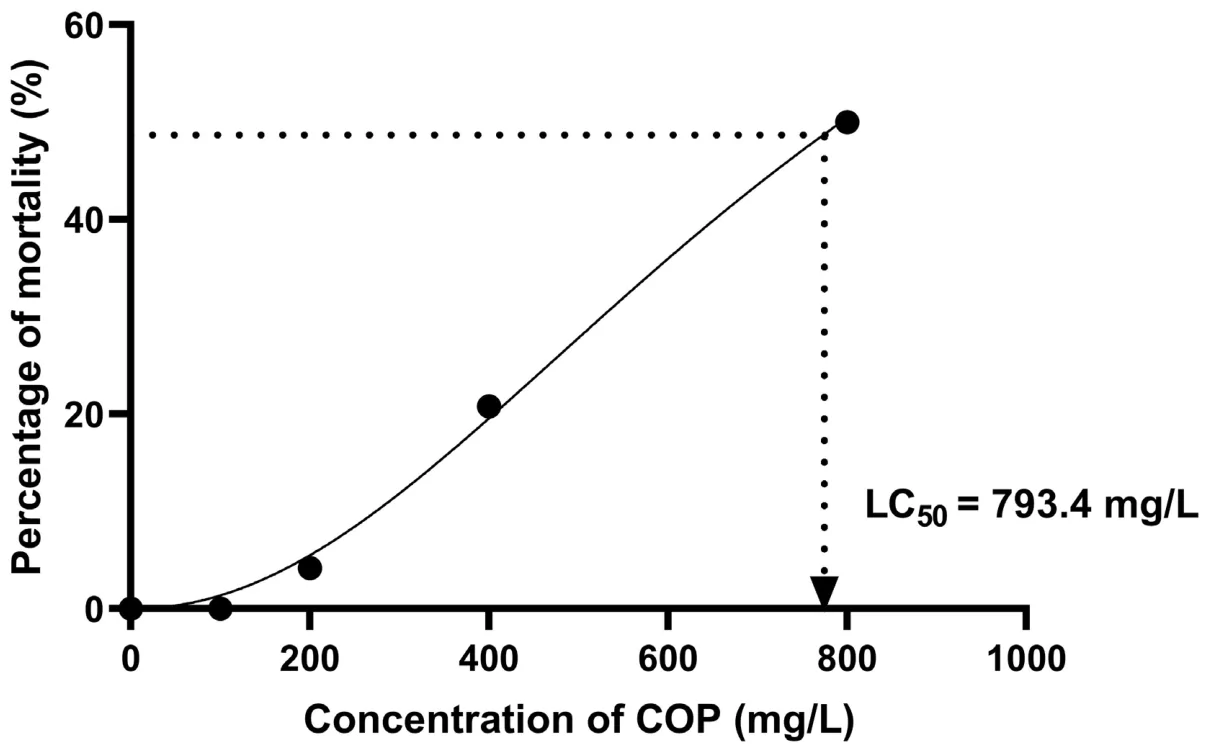
Acute toxicity curve and 96 h LC_50_ determination of COP in *Betta splendens*. Mortality percentages were fitted using a non-linear four-parameter dose–response model. The dashed line indicates a 96 h LC _50_ value of 793.4 mg/L. Data points represent *n* = 24 fish per group.

The mortality rate of *Betta splendens* following exposure to different COP is presented in Table 2. During 96 h of exposure period, the highest mortality was exhibited at the concentration of 800 mg/L (50%), followed by the concentration of 400 mg/L (20.8%) and 200 mg/L (4.17%), respectively. Based on a non-linear four-parameter dose–response model, the calculated 96 h LC_50_ value for COP in *Betta splendens* was 793.4 mg/L (R^2^ = 0.997, 95% CI = [736.6–866.8] mg/L) (Figure 1). Fish exposed to the highest concentration showed distinct clinical signs and behavioral abnormalities, including increased opercular movement, restlessness, rapid surface breathing, and skin irritation. Based on the results of the toxicity assay, where the 100 mg/L concentration showed no significant mortality or behavioral changes compared to the control group, this dose was excluded from subsequent molecular and histological analyses. Consequently, downstream assays including oxidative stress markers, mRNA expression, and histopathology were performed on the 0, 200, 400, and 800 mg/L groups to characterize the dose-dependent metabolic stress response.

### 3.2. Effect of COP Exposure on Oxidative Stress Biomarkers

**Figure 2:**
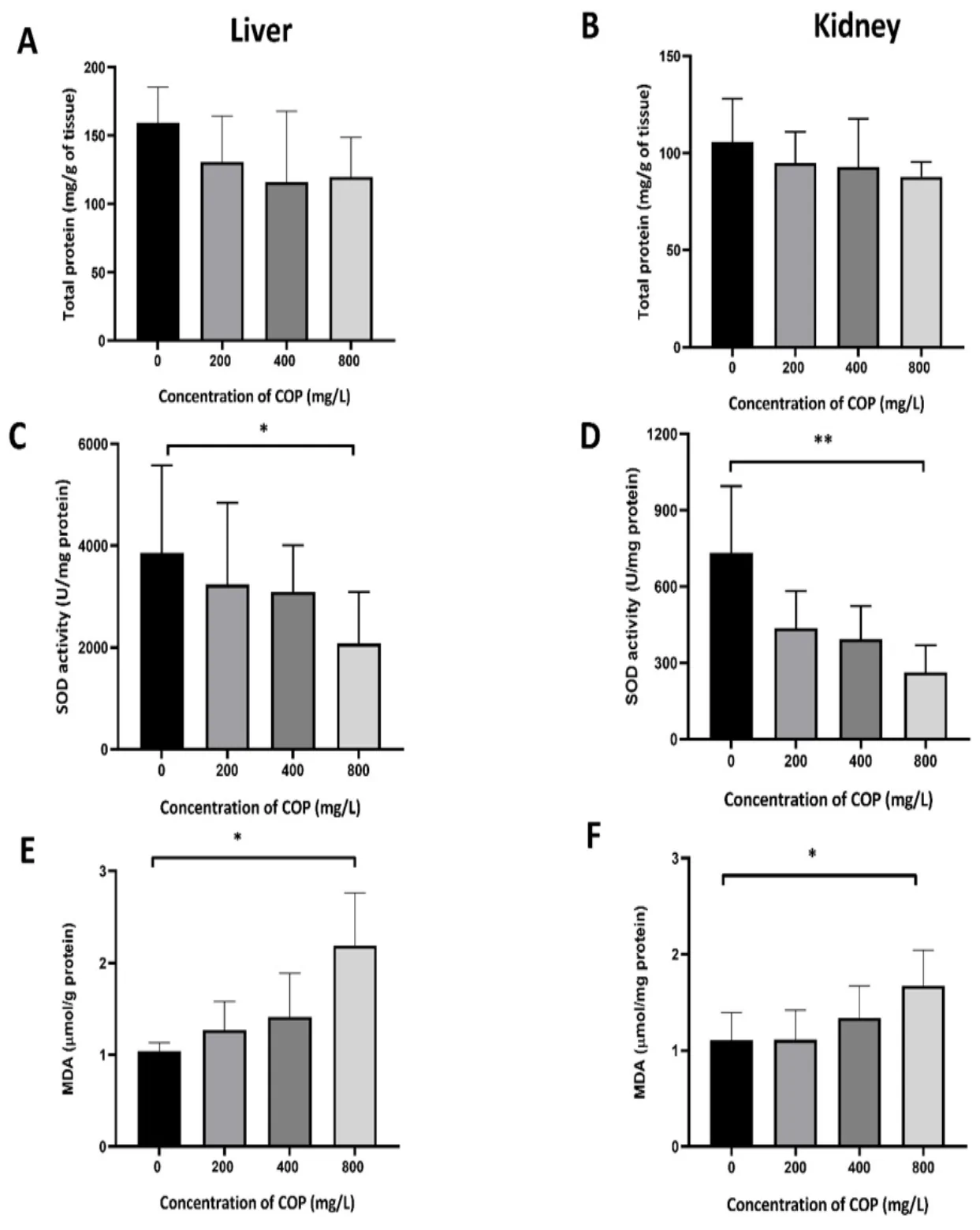
Oxidative stress biomarkers in *Betta splendens* following 96 h COP exposure. Data represent (**A**,**B**) total protein level, (**C**,**D**) superoxide dismutase (SOD) activity, and (**E**,**F**) malondialdehyde (MDA) levels in liver and kidney tissues, respectively. Values are expressed as means ± SD (*n* = 5 independent replicates). Group comparisons were analyzed using the Kruskal–Wallis test followed by Dunn’s post hoc test (* *p* < 0.05, ** *p* < 0.01, vs. control). A linear dose–response relationship across exposure groups was evaluated using a post hoc trend analysis: SOD activity in liver (*p_trend_* = 0.038), SOD activity in kidney (*p_trend_* = 0.0002), MDA level in liver (*p_trend_* = 0.009), and MDA level in kidney (*p_trend_* = 0.0096).

In fish, the liver and kidney have antioxidant defense systems that play a critical role in combating oxidative stress triggered by environmental pollutants. SOD acts as a critical first-line antioxidant enzyme by converting toxic superoxide radicals into hydrogen peroxide, which is subsequently neutralized by downstream enzymes to maintain cellular health in vital organs [34]. MDA is a widely used biomarker for assessing oxidative stress and lipid peroxidation induced by environmental pollutants [35]. To investigate the acute effects of COP exposure on the antioxidant status, the total protein, antioxidant enzyme activity, and MDA level in liver and kidney tissues were measured and are shown in Figure 2. We found that the total protein levels in both hepatic and renal tissues remained statistically unaltered across exposure concentrations (*p* > 0.05; Figure 2A,B). Superoxide dismutase (SOD) activity was significantly reduced at COP concentrations of 800 mg/L in the liver (*p* = 0.03) and kidney (*p* = 0.002; Figure 2C,D). Furthermore, post hoc trend analysis revealed a significant linear dose-dependent reduction in SOD activity for both hepatic (*p_trend_* = 0.038) and renal (*p_trend_* = 0.0002) tissues compared to the control. Concurrently, a significant elevation in malondialdehyde (MDA) levels was observed in the liver (*p* = 0.025, *p_trend_* = 0.009), and kidney (*p* = 0.037, *p_trend_* = 0.0096) following 96 h of exposure to 800 mg/L COP relative to control (Figure 2E,F). This increase indicates that COP exposure triggers significant lipid-phase cellular membrane damage.

### 3.3. Expression of Genes Related to Metabolic Stress Response in Target Organs

**Figure 3:**
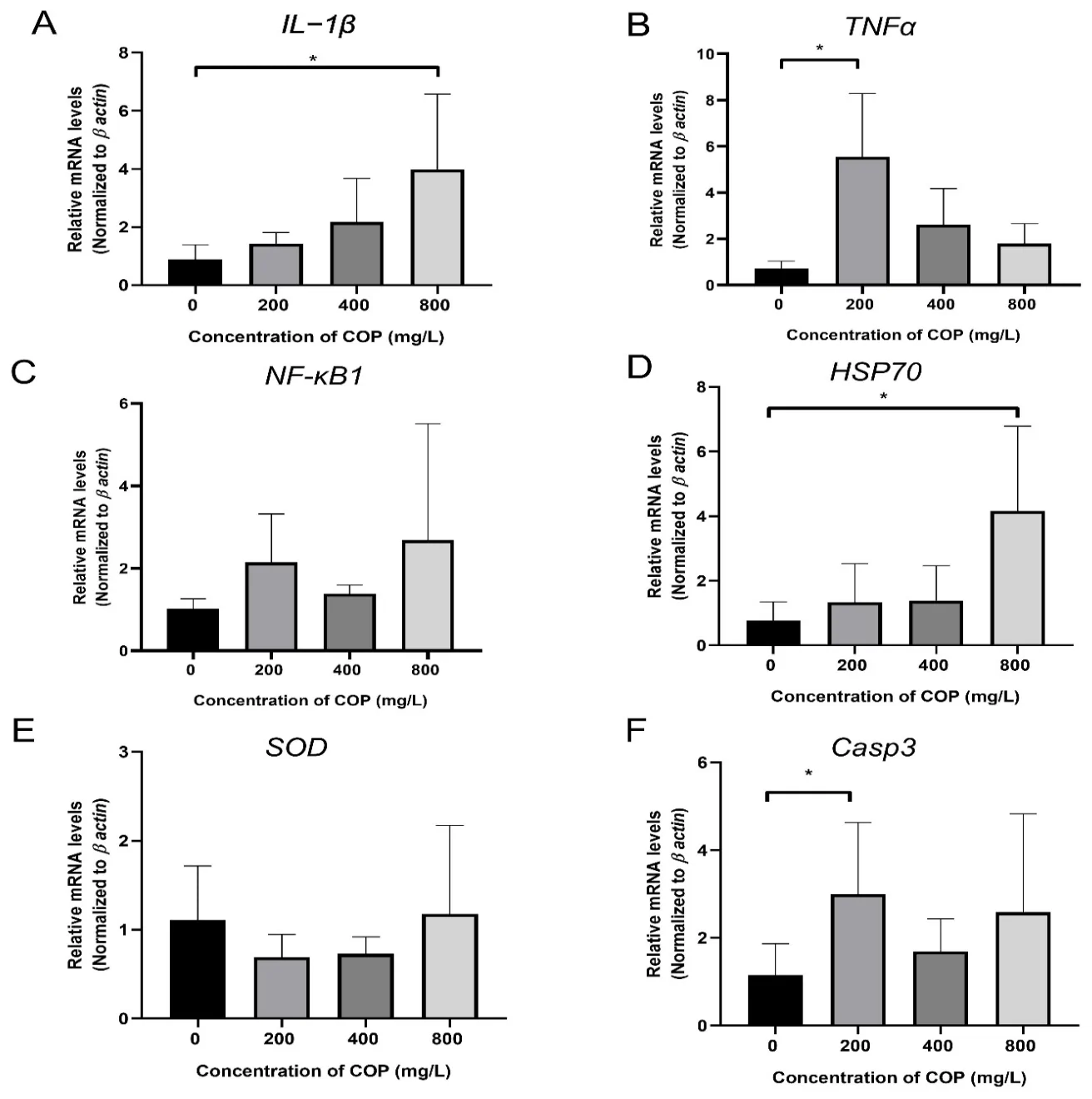
Relative mRNA expression levels of (**A**) *IL*-1*β*, (**B**) *TNF-α*, (**C**) *NF-κB*1, (**D**) *HSP*70, (**E**) *SOD*, and (**F**) *Casp*3 in liver of *Betta splendens* exposed to various levels of COP concentration (0–800 mg/L) for 96 h. The relative mRNA abundance was normalized to the endogenous reference gene *β-actin*. Data are expressed as means ± SD of five independent replicates (*n* = 5). Statistical differences between treatment groups and the control group were evaluated using one-way ANOVA followed by Dunnett’s post hoc test (* p < 0.05). Overall concentration-dependent linear trends across exposure groups were evaluated using linear regression analysis (*p_trend_* < 0.05).

The toxic impact of COP at the transcriptomic level was evaluated by analyzing functional target genes associated with pro-inflammatory signaling, cellular stress, antioxidant defense, and apoptosis in liver and kidney. Overall, exposure to COP significantly altered gene expression profiles in both tissues, with key markers exhibiting distinct concentration-dependent linear trends (*p_trend_* < 0.05). Both *IL*-1*β* and *TNF-α* serve as primary indicators of an inflammatory response, which is frequently triggered by chemical exposure or tissue injury in aquatic species [36,37]. In the liver, the pro-inflammatory cytokine *IL*-1*β* showed a significant dose-dependent linear upregulation (*p_trend_* = 0.049), yielding significant upregulation at the highest concentration of 800 mg/L (*p* = 0.036) (Figure 3A). Conversely, *TNF-α* expression responded at a lower concentration (200 mg/L), showing significant upregulation (*p* = 0.019), which subsequently plateaued at higher concentrations (400–800 mg/L) (Figure 3B). Concurrently, a general upward trend in *NF-κB*1 expression was observed (Figure 3C). Regulating cellular stress, *HSP*70 displayed a significant positive dose–response trend (*p_trend_* = 0.03), with marked upregulation at 800 mg/L (*p* = 0.028) (Figure 3D).

**Figure 4:**
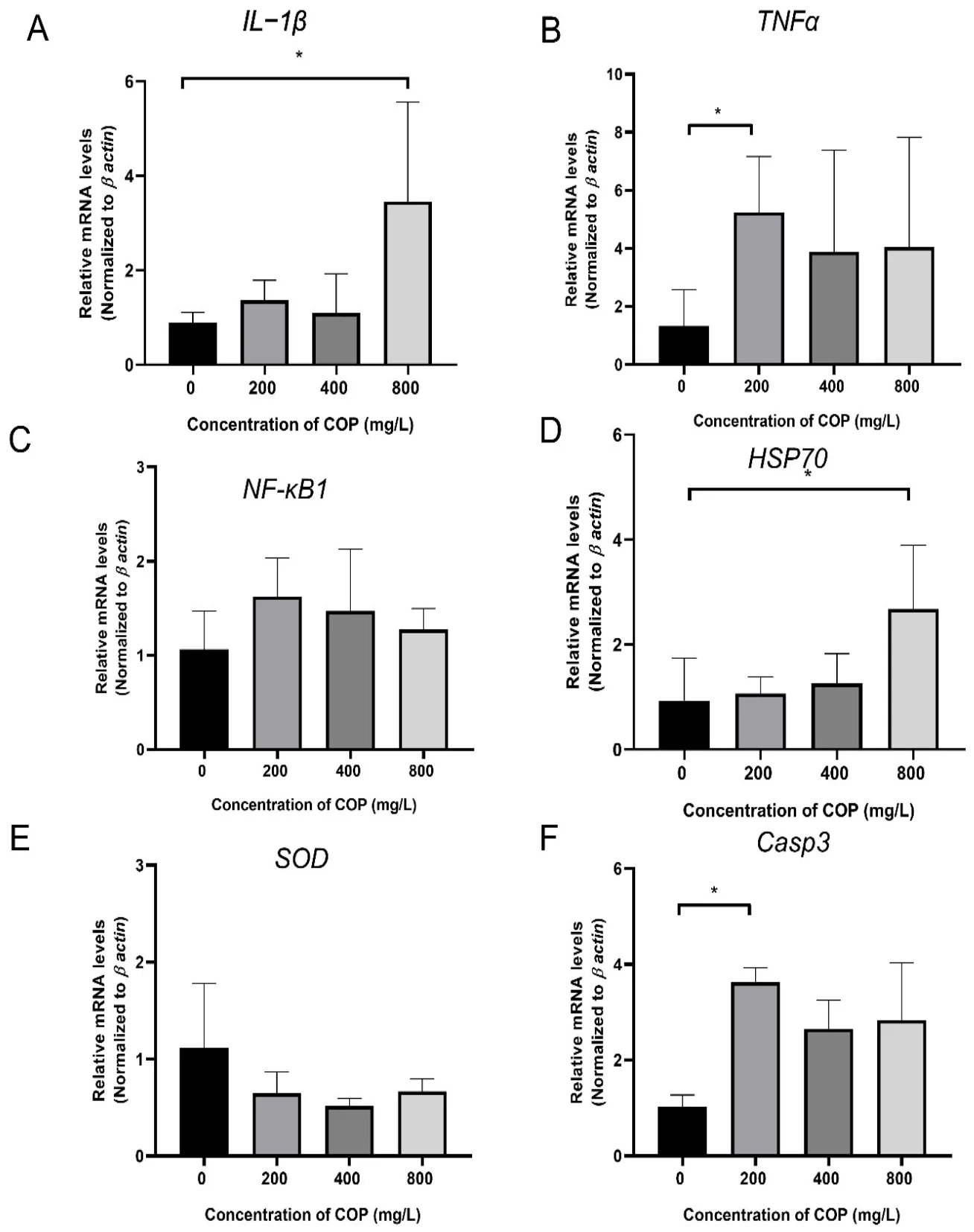
Relative mRNA expression levels of (**A**) *IL*-1*β*, (**B**) *TNF-α**,*** (**C**) *NF-κB*1***,*** (**D**) *HSP*70***,*** (**E**) *SOD*, and (**F**) *Casp*3 in kidney of *Betta splendens* exposed to various levels of COP concentration (0–800 mg/L) for 96 h. The relative mRNA abundance was normalized to the endogenous reference gene *β-actin*. Data are expressed as means ± SD of five independent replicates (*n* = 5). Statistical differences between treatment groups and the control group were evaluated using one-way ANOVA followed by Dunnett’s post hoc test (* p < 0.05). Overall concentration-dependent linear trends across exposure groups were evaluated using linear regression analysis (*p_trend_* < 0.05).

In the kidney, the transcriptomic profile closely mirrored hepatic patterns. Renal *IL*-1*β* demonstrated a significant linear dose-dependent increase (*p_trend_* = 0.049) reaching peak induction at 800 mg/L (*p* = 0.033) (Figure 4A). *TNF-α* expression was significantly upregulated at 200 mg/L (*p* = 0.048) and remained sustained across higher exposure groups (Figure 4B), while *NF-κB*1 exhibited a mild upregulatory response (Figure 4C). Cellular stress in renal tissue was strongly concentration-dependent, as evidenced by a highly significant dose–response trend for *HSP*70 (*p_trend_* = 0.01), peaking at 800 mg/L (*p* = 0.028) (Figure 4D). Additionally, the apoptotic marker *Casp*3 exhibited a significant positive linear trend across exposure groups (*p_trend_* = 0.03), beginning with marked upregulation at 200 mg/L (*p* = 0.019) and remaining elevated through 800 mg/L (Figure 4F). Other tested genes did not show statistically significant dose-dependent linear trends (*p_trend_* > 0.05).

### 3.4. Histopathological Analysis

**Figure 5:**
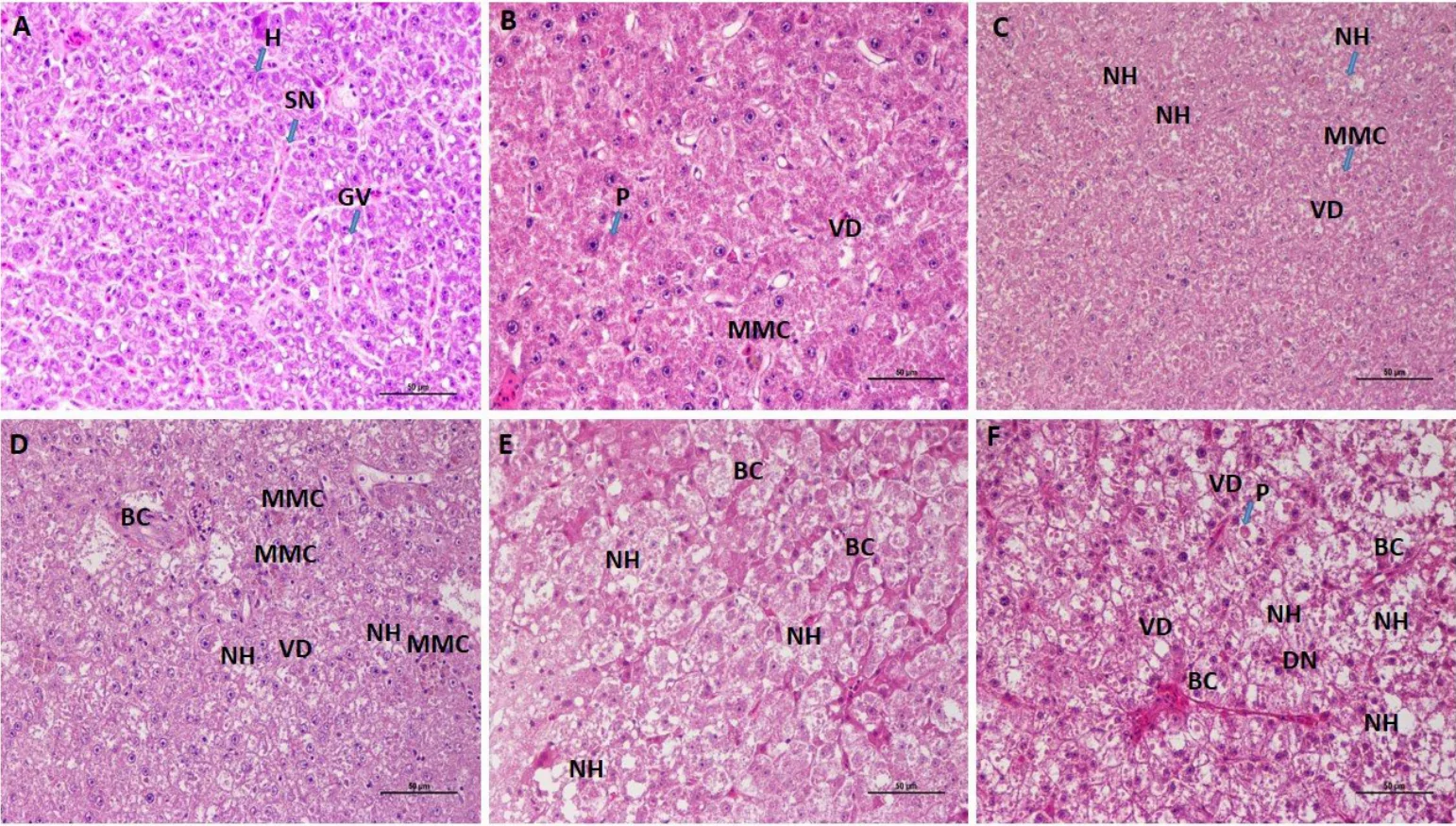
Microphotographs of hematoxylin and eosin (H&E) staining reveal the histopathological changes in the liver of *Betta splendens* observed after various concentrations of COP exposures at 96 h. (**A**) Liver sections of the control group, showing normal liver features with hepatocytes (H), sinusoids (SN), and glycogen vacuolation (GV). (**B**–**F**) Fish exposed to COP for 96 h at concentrations of 200 mg/L (**B**), 400 mg/L (**C**), and 800 mg/L (**D**–**F**), showing vacuolar degeneration (VD), melanomacrophage (MMC) aggregation, blood congestion (BC), degenerated nuclei (DN), pyknosis (p), and necrotic hepatocytes with fading nuclei (karyolysis) (NH). Scale bars correspond to 50 µm at 400× magnification.

To assess structural organ damage, histopathological impacts of COP exposure were evaluated in the liver and kidney of *Betta splendens*. Tissue alterations were semi-quantitatively scored on a scale from 0 to 3 (0: no lesion, 1: mild, 2: moderate, 3: severe) with total lesion scores representing the overall organ damage index (Table 3 and Table S1). The liver is crucial organ that filters blood and drives the body’s metabolic processes. Following 96 h of exposure, a clear dose-dependent increase in tissue damage was observed. Control specimens exhibited intact hepatic architecture with only mild, diffuse cytoplasmic vacuolation in localized hepatocytes, representing physiological glycogen storage (Figure 5A). In contrast, COP-exposed groups demonstrated progressive, dose-dependent structural deterioration (Figure 5B–F). At 200 mg/L, mild vacuolar degeneration and melanomacrophage (MMC) aggregation were initiated (Figure 5B). Higher exposure concentrations including 400 mg/L (Figure 5C) and 800 mg/L (Figure 5D–F) induced severe parenchymal disruption, characterized by variable size with open chromatin alongside pyknotic, intensely basophilic shrunken nuclei, prominent blood congestion and extensive hepatocyte necrosis. Consequently, total hepatic lesion scores increased significantly at 400 mg/L (1.75 ± 0.25, *p* = 0.03) and peaked at 800 mg/L (2.35 ±0.14, *p* = 0.0003) compared to the control group (Table 3).

**Figure 6:**
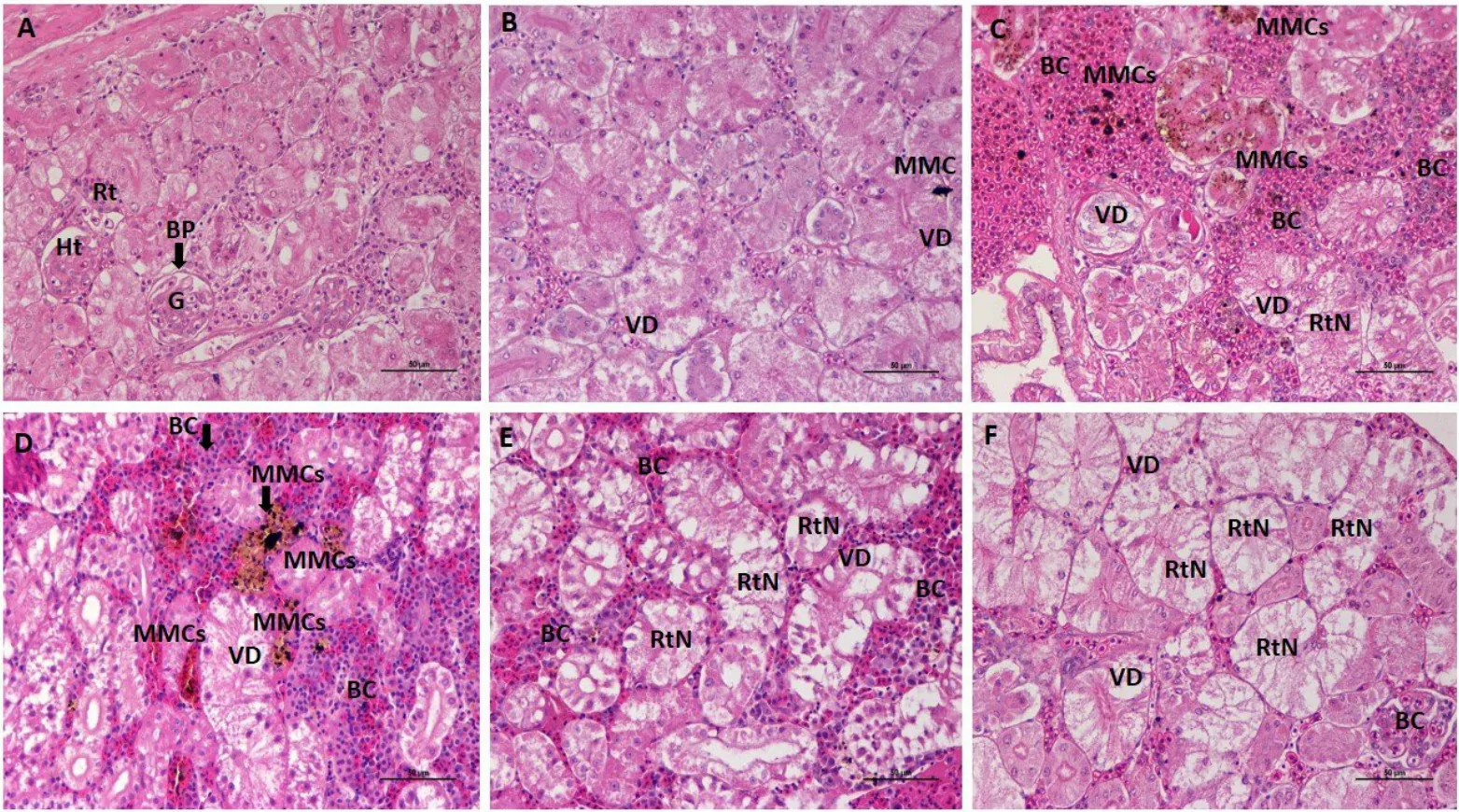
Microphotographs of H&E staining reveal histopathological changes in the kidney of *Betta splendens* observed after various concentrations of COP exposures at 96 h. (**A**) Kidney sections of the control group, showing normal renal features with numerous renal tubules (Rt), glomerulus (G), hematopoietic tissue (Ht), and Bowman’s capsules (BP). (**B**–**F**) Fish exposed to COP for 96 h at concentrations of 200 mg/L (**B**), 400 mg/L (**C**), and 800 mg/L (**D**–**F**), showing vacuolar degeneration (VD), melanomacrophage (MMC) aggregation, blood congestion (BC), and renal tubular necrosis (RtN). Scale bars correspond to 50 µm at 400× magnification.

In fish, the kidneys serve as vital metabolic and excretory organs that regulate blood pH, maintain osmotic balance, and filter out metabolic waste. After 96 h of exposure, unexposed control fish displayed typical renal morphology with structurally intact glomeruli, Bowman’s capsules, and renal tubules (Figure 6A). Following 96 h of COP exposure, renal tissues developed marked pathological lesions (Figure 6B–F). While mild alterations were recorded at 200 mg/L (Figure 6B), exposure to 400 mg/L (Figure 6C) and 800 mg/L (Figure 6D–F) led to significant accumulation of tubular vacuolar degeneration, MMC aggregation, vascular congestion and widespread tubular necrosis-dependent elevation at 400 mg/L (2.22 ± 0.21, *p* = 0.02) and 800 mg/L (2.65 ± 0.22, *p* = 0.0004) relative to controls (Table 3).

### 3.5. COP Concentration Analysis in Experimental Water

The determination of the actual COP concentrations in the water samples after exposure was performed using a 6546 LC-Q-TOF/MS system (Agilent Technologies, CA, USA). The analytical method was validated, demonstrating good linearity (R^2^ ≥ 0.998); high recovery rates ranged from 97.08% to 107.21%, which fell within the acceptable limits, demonstrating the reliability and accuracy of the method. The LOD and LOQ values for COP were 0.07 and 0.50 µg/L, respectively. Following a 96 h exposure period, the measured concentrations of COP at each respective exposure level (100, 200, 400, and 800 mg/L) were 98.9 ± 1.58, 141.34 ± 11.58, 376.5 ± 0.56, and 695.1 ± 13.34 mg/L. These results verify that the COP concentrations remained close to the designated targets throughout the 96 h exposure period.

## 4. Discussion

Recent ecotoxicological studies have extensively investigated the impacts of EPCs on standard fish models, particularly the zebrafish [38,39]. However, these standard models may not fully reflect the unique metabolic limitations of labyrinth fish. *Betta splendens*, a member of the Betta genus, typically inhabits slow-moving or stagnant tropical waters where agricultural and urban wastewater runoff heavily accumulate [40], making them an ecologically vital model for localized contamination risks. Chemicals of emerging concern, particularly anticancer drugs, have drawn significant scientific attention due to their environmental stability. Additionally, the potential effects of these parent compounds or their derivatives on non-target organisms are a major concern. Among these EPCs, COP has been the most frequently reported in terms of environmental persistence [41,42] and aquatic toxicity [43,44,45]. As it is known, COP is one of the most widely used chemotherapeutic and immunosuppressive drugs worldwide. Belonging to the class of alkylating agents, it can induce hepatic oxidative stress, and renal damage in humans and animals [46]. Fish absorb COP from the surrounding water which is then distributed via the bloodstream to key metabolic organs. Due to their unique anatomical structures, high blood perfusion, and specialized physiological roles, the liver and kidneys are highly susceptible to xenobiotic-induced toxicities [47]. The liver is the principal site of COP bioaccumulation during metabolic processing and detoxification. Here, the pollutants disrupt crucial enzyme pathways and trigger severe oxidative stress [48]. Concurrently, the kidneys suffer structural and cellular damage during the filtration and osmoregulation processes [33].

In this study, the acute toxicity of COP in male *Betta splendens* was evaluated under controlled conditions across a concentration range of 0 to 800 mg/L over a 96 h exposure period. The acute toxicity of COP has been documented across several aquatic models. For instance, the 24 h LC_50_ values for *Ceriodaphnia dubia* and *Thamnocephalus platyurus* were reported at 987 mg/L and 1396 mg/L, respectively [49], while the LC_50_ value for *Daphnia magna* exceeds 1000 mg/L [50]. In vertebrate models, the 96 h LC_50_ values for zebrafish embryo and larva were documented at 1306 mg/L [14]. However, there remains a critical knowledge gap regarding the ecotoxicological sensitivity of local fish species endemic to Thailand to COP contaminants. Interestingly, the results of this study reveal an increased susceptibility in this native labyrinth fish compared to previous studies. Specifically, the 96 h LC_50_ value for COP in *Betta splendens* was determined to be 793.4 mg/L. It is highly probable that the observed differences in tolerance are due to the undeveloped metabolic systems and protective chorion of the embryonic forms, which protect zebrafish embryo and larva. Conversely, the susceptibility of adult fish is probably attributed to their fully active cytochrome P450 enzymes, which may metabolize COP into its toxic metabolites [51,52].

COP exposure induced severe oxidative stress throughout all tissues, particularly affecting metabolic organs. The excessive generation of ROS overwhelmed cellular defenses. SOD acts as the primary defense against the toxic effects of superoxide radicals. In response to environmental stress, its activity often increases initially to scavenge superoxide radicals [53]. However, prolonged or severe exposure can lead to a subsequent decline in SOD activity as the enzyme is exhausted [54]. This oxidative stress triggered widespread lipid peroxidation across various tissues, which was confirmed by a dose-dependent increase in MDA. As a byproduct of polyunsaturated fatty acid decomposition, MDA is a classic indicator of oxidative damage to cell membranes. In aquatic toxicology, it serves as a vital biomarker for assessing how environmental pollutants and toxins induce oxidative stress in fish [55]. In this study, a 96 h exposure to COP at the concentration 800 mg/L significantly decreased SOD activity and increased MDA levels in liver and kidney tissues of *Betta splendens*. This shift indicates that COP triggers severe cellular membrane damage, driven by ROS attacking hepatic and renal lipid structures. These findings are in strong agreement with previous studies. For instance, Sahu et al. reported that a high dose of COP (200 mg/kg) overwhelms hepatic and renal antioxidant defenses, causing profound cellular damage in rats [56]. Similarly, a 100 mg/kg COP treatment has been shown to induce severe oxidative stress, which subsequently triggers a hepatic inflammatory response in Nile tilapia [57].

Previous studies have demonstrated that pharmaceutical contaminants interfere with immune homeostasis in aquatic organisms [58]. In the present study, COP exposure significantly increased the expression levels of *IL*-1*β* in a threshold-dependent manner in both the liver and kidney. *IL*-1*β* is a potent pro-inflammatory cytokine in fish that serves as the master regulator of the acute immune response to tissue injury. This obvious upregulatory profile demonstrates that a COP concentration of 800 mg/L induces severe cellular and tissue damage in both the hepatic and renal systems. Furthermore, a low concentration of COP (200 mg/L) induced *TNF-α* expression, a primary cytokine that regulates inflammatory responses. This finding suggested that *TNF-α* acts as a sensitive cytokine early-warning system in *Betta splendens* to COP-induced tissue injury. Concurrently, *NF-κB*1 transcription displayed a non-significant upward trend without reaching statistical significance. Typically, the key nuclear transcription factor NF-κB is activated at the post-translational level via the phosphorylation and degradation of inhibitor of nuclear factor kappa B (IκB) protein, allowing it to translocate into the nucleus and drive the transcription of downstream pro-inflammatory targets such as *IL*-1*β* and *TNF-α* under pathological conditions [59]. Based on our findings, a possible molecular mechanism linking COP to organ damage begins with its hepatic biotransformation into active cytotoxic metabolites. This metabolic activation induces severe oxidative stress through the generation of massive amounts of ROS, thereby triggering the degradation of inhibitory proteins and causing the NF-κB complex to translocate into the cell nucleus to directly upregulate pro-inflammatory target genes such as *IL*-1*β* and *TNF-α.* This triggers both systemic and localized inflammatory responses, leading to tissue damage, and cellular apoptosis in vital organs. The previous literature has established that COP upregulates these key inflammatory mediators [60,61]. Consistent with those reports, our findings collectively support the assumption that COP acts as a potent chemical stressor, driving acute tissue damage and triggering localized inflammatory responses in fish [57]. However, as NF-κB primarily signaling operates through post-translational modification and nuclear translocation rather than direct transcriptional upregulation of its mRNA, evaluating gene expression alone may not fully capture pathway activity. To definitively confirm the functional activation of the NF-κB signaling cascade under COP exposure, future studies should quantify total and phosphorylated NF-κB protein levels supplemented by nuclear localization assays and pathway-specific inhibitor experiments.

In aquatic organisms, the upregulation of *HSP*70 serves as a bioindicator for monitoring cellular stress triggered by environmental pollutants. Across various fish species, these molecular chaperones play a vital role in maintaining cellular balance and enhancing immune defenses as an immediate defense mechanism against environmental stressors [62]. Simultaneously, SOD acts as a crucial first line of antioxidant defense by scavenging superoxide radicals to prevent cellular damage. In this study, exposure to COP triggered a significant increase in *Hsp*70 expression in the liver and kidney, which was accompanied by a non-significant decreasing trend in *SOD* expression. However, *SOD* mRNA levels in the liver displayed a non-significant compensatory trend back to baseline control levels at the highest COP concentration. This pattern suggests a dose-dependent compensatory attempt against severe accumulation of ROS, prompting the upregulation of antioxidant genes to mitigate oxidative stress. Interestingly, while SOD mRNA levels in both tissues showed a non-significant upward trend at 800 mg/L COP, actual SOD protein levels measured by ELISA decreased significantly. This indicates that acute chemical toxicity temporarily overwhelms translational machinery despite potential transcriptional attempts. Furthermore, severe cellular stress accelerates protein degradation. Under these compromised conditions, the overexpression of *Hsp*70 serves as an adaptive mechanism to preserve cells from oxidative stress-induced damage. However, the prolonged stress signaling overwhelms the fish’s antioxidant defenses; it causes severe oxidative stress that results in DNA, lipid, and protein damage [63]. This persistent stress directly activates pro-apoptotic signaling pathways, leading to an elevation of *Casp*3 activity, the key enzyme that executes apoptosis [64]. Ultimately, the continuous overproduction of *HSP*70 and subsequent apoptotic signaling induce severe tissue damage, such as cell swelling and necrosis [65], which was confirmed by the upregulated *Casp*3 expression.

Evaluating the acute toxicity of EPCs is critical during accidental environmental releases. The data from these studies address the onset of morphological, behavioral, and metabolic alterations as well as molecular responses within aquatic organisms [66]. To validate these findings, liver and kidney biopsies can be used as bioindicators of systemic health and environmental pollution levels. In the present study, the histopathological impacts on the liver and kidney were evaluated following 96 h of COP exposure. As the primary organ driving blood filtration and metabolic processing, the liver exhibited a distinct concentration-dependent response. At the lowest concentration of COP (200 mg/L), the liver exhibited mild fatty degeneration and MMC aggregation. At higher concentrations (400 and 800 mg/L), pathological changes intensified into severe parenchymal disruption characterized by open chromatin, nuclear pyknosis, prominent blood congestion, extensive cellular degeneration, and widespread hepatocyte necrosis. Consequently, total hepatic lesion scores increased significantly at 400 mg/L and peaked at 800 mg/L compared to controls. These findings corroborate the previous literature that exposure to COP induces hepatic injury and escalates mortality rates in Nile tilapia [67] and zebrafish [19]. Parallel to the liver, the kidney displayed significant histopathological damage across the various COP-exposed fish groups, with the most severe abnormalities manifesting at the highest concentration of 800 mg/L. These renal histological changes included congestion of the renal blood vessels, MMC aggregation, and vacuolar degeneration, culminating in widespread tubular necrosis. This structural decay was reflected in total renal lesion scores, which rose significantly at 400 mg/L and 800 mg/L relative to controls. The concurrent destruction of both hepatic and renal architectures directly explains the marked upregulation of *Casp*3 expression across both tissues, indicating that COP-induced toxicity actively triggered widespread apoptosis. Furthermore, these concurrent hepatic and renal alterations closely reflect the systemic toxic effects reported in mammalian models [68,69]. Overall, the severe structural changes observed in both the liver and kidneys of COP-exposed *Betta splendens* demonstrate profound functional impairment, which ultimately disrupts the vital metabolic mechanisms.

## 5. Conclusions

This study demonstrates that waterborne COP exposure induces severe toxicological effects in *Betta splendens*. These impacts are clearly evidenced by intensified oxidative stress, alterations in gene expression, and localized histopathological changes within the liver and kidney. The significant stimulation of enzymatic antioxidants like SOD, alongside marked lipid peroxidation indicates a distinct failure of the fish’s antioxidant defense system to neutralize excessive ROS generation, ultimately triggering cellular apoptosis. Using the *Betta splendens* as a highly relevant local model obviously demonstrates how EPCs threaten Thailand’s native aquatic biodiversity. To better facilitate the risk assessment of this class of emerging contaminants, further investigation into the specific mechanisms of COP and chemotherapy drug cocktails within non-target aquatic species remains essential to advance ecotoxicological risk assessments. Moreover, the characterizing the pharmacokinetics and biotransformation pathways of COP in *Betta splendens* is required to fully elucidate its systemic toxicity mechanisms. Ultimately, this study highlights the necessity of targeted environmental monitoring to safeguard Thailand’s native freshwater species.

## Figures and Tables

**Table 1:** Primer sequences used for this study.

Gene	Primer Sequences	Length (bp)	References/Accession Number
*IL*-1*β*	F: 5′-GGAGGTTGTGGAGGACAAA-3′ R: 5′-CTCTGTCCGGTGCTGATGTA-3′	150	XM_029164102.3 [31]
*TNFα*	F: 5′-CCTTGTGTTCCACAGACACG-3′ R: 5′-GTGGGACTGGTCAACCTGAT-3′	172	XM_029166845.3 [31]
*NF-κB*1	F: 5′-GGCCAGGTAATGCGTCTACA-3′ R: 5′-GAAATCCCTGTGAGGCCAGT-3′	146	XM_029132477.3
*HSP*70	F: 5′-GGGAGCTGAACAAGAGCATC-3′ R: 5′-ATGGTGGTGTTCCGTTTGAT-3′	190	XM_029157549.2 [32]
*SOD*	F: 5′-GCAGCCATTGCAACAACGTA-3′ R: 5′-ATGGTGACGTTGCCATTCCT-3′	232	XM_029147413.3
*Casp*3	F: 5′-GATGGGAGCTGGTTTGTCCA-3′ R: 5′-TGAGAACTGGAGCCCTCCTT-3′	128	XM_055503663.1
*β-actin*	F: 5′-AGGCTGTGCTGTCCCTGTAT-3′ R: 5′-GAAGGAGTAGCCACGCTCTG-3′	200	[32]

**Table 2:** Mortality rate of *Betta splendens* exposed to different concentrations of COP (*n* = 24, each concentration).

Time (h)	COP Concentration (mg/L)				
	0	100	200	400	800
24 h	0	0	0	0	0
48 h	0	0	0	0	0
72 h	0	0	0	0	0
96 h	0	0	1	5	12
Total number of deaths	0	0	1	5	12
Total percentage of fish mortality (%)	0	0	4.17	20.8	50

**Table 3:** Semi-quantitative evaluation of histological alterations in the liver and kidney of *Betta splendens* exposed to different concentrations of COP.

Organ	Control	COP 200 mg/L	COP 400 mg/L	COP 800 mg/L
Liver histopathology				
Total lesion score	0.3 ± 0.11	0.65 ± 0.22	1.75 ± 0.25 *	2.35 ± 0.14 ***
Kidney histopathology				
Total lesion score	0.2 ± 0.13	0.6 ± 0.29	2.2 ± 0.21 *	2.65 ± 0.22 ***

Data are presented as means ± SD (*n* = 5 per group). Histological alterations were graded on a semi-quantitative scale ranging from 0 to 3 (0: no lesion; 1: mild; 2: moderate; 3: severe). The total lesion score represents the overall tissue damage index per organ on a scale (maximum score = 3). Statistical differences between treatment groups and the control group were evaluated using the non-parametric Kruskal–Wallis test followed by Dunn’s post hoc test. Asterisks denote statistically significant differences compared to the control group (* *p* < 0.05 and *** *p* < 0.001).

## Data Availability

Data that support the findings of this study are available from the corresponding author upon reasonable request.

## Supplementary Materials

The following supporting information can be downloaded at: https://www.mdpi.com/article/10.3390/toxics14080700/s1, Table S1: Histopathological lesion indices and tissue-level damage scores in the liver and kidney of *Betta splendens* under acute cyclophosphamide (COP) exposure.
