# Supplementary material for: Acute Toxic Impact of Cyclophosphamide on the Metabolic Organs of Siamese Fighting Fish (Betta splendens)
Source: Toxics. 2026 Aug 7;14(8):700. doi: 10.3390/toxics14080700 (PMC13517365; doi:10.3390/toxics14080700)
Supplement: Supplementary file 1 [file toxics-14-00700-s001.zip › toxics-4446912-supplementary.pdf]

Table S1. Histopathological lesion indices and tissue-level damage scores in the liver and kidney of *Betta splendens* under acute cyclophosphamide (COP) exposure

**Organ / Histological Alteration**	Control	COP 200 mg/L	COP 400 mg/L	COP 800 mg/L
**Liver histopathology**				
Vacuolar degeneration	0.3±0.11	0.45±0.11	0.6±0.14	0.75±0.11
MMCs	0	0.2±0.11	0.45±0.11	0.45±0.11
Blood congestion	0	0	0.25±0.0	0.5±0.0
Necrosis	0	0	0.45±0.21	0.7±0.11
Total lesion score	0.3±0.11	0.65±0.22	1.75±0.25*	2.35±0.14***
**Kidney histopathology**				
Vacuolar degeneration	0.2±0.13	0.25±0.18	0.55±0.11	0.7±0.11
MMCs	0	0.15±0.14	0.6±0.14	0.65±0.22
Blood congestion	0	0.2±0.11	0.55±0.11	0.6±0.14
Tubular necrosis	0	0	0.5±0.0	0.7±0.11
Total lesion score	0.2±0.13	0.6±0.29	2.2±0.21*	2.65±0.22***

Data are presented as means ± SD (n = 5 per group). Individual histological alterations were graded on a semi-quantitative scale ranging from 0 to 3 (0: no lesion; 1: mild; 2: moderate; 3: severe). The total lesion score represents the overall tissue damage index per organ on a scale (maximum score = 3). Statistical differences between treatment groups and the control group were evaluated using the non-parametric Kruskal-Wallis test followed by Dunn's post-hoc test. Asterisks denote statistically significant differences compared to the control group (*$p < 0.05$ and ***$p < 0.001$). MMC: Melanomacrophage aggregation
